# Characterization and In Vivo Anti-Inflammatory Efficacy of Copal (*Dacryodes peruviana* (Loes.) H.J. Lam) Essential Oil

**DOI:** 10.3390/plants11223104

**Published:** 2022-11-15

**Authors:** Lupe Carolina Espinoza, Eduardo Valarezo, María José Fábrega, María José Rodríguez-Lagunas, Lilian Sosa, Ana Cristina Calpena, Mireia Mallandrich

**Affiliations:** 1 Departamento de Química, Universidad Técnica Particular de Loja, Loja 1101608, Ecuador; 2 Institute of Nanoscience and Nanotechnology (IN2UB), University of Barcelona, 08028 Barcelona, Spain; 3 Department of Experimental and Health Sciences, Parc of Biomedic Research of Barcelona, Pompeu Fabra University, 08003 Barcelona, Spain; 4 Department of Biochemistry and Physiology, Faculty of Pharmacy and Food Sciences, University of Barcelona, 08028 Barcelona, Spain; 5 Nutrition and Food Safety Research Institute (INSA-UB), 08921 Santa Coloma de Gramenet, Spain; 6 Pharmaceutical Technology Research Group, Faculty of Chemical Sciences and Pharmacy, National Autonomous University of Honduras (UNAH), Tegucigalpa 11101, Honduras; 7 Department of Pharmacy, Pharmaceutical Technology and Physical Chemistry, Faculty of Pharmacy and Food Sciences, University of Barcelona, 08028 Barcelona, Spain

**Keywords:** inflammation, essential oil, *Dacryodes peruviana*, α-phellandrene, limonene

## Abstract

Essential oils are natural aromatic substances that contain complex mixtures of many volatile compounds frequently used in pharmaceutical and cosmetic industries. *Dacryodes peruviana* (Loes.) H.J. Lam is a native species from Ecuador whose anti-inflammatory activity has not been previously reported, thus the aim of this study was to evaluate the anti-inflammatory activity of *D. peruviana* essential oil. To that end, essential oil from *D. peruviana* fruits was isolated by hydrodistillation and characterized physically and chemically. The tolerance of the essential oil was analyzed by cytotoxicity studies using human keratinocytes. The anti-inflammatory activity was evaluated by an arachidonic acid-induced edema model in mouse ear. The predominant compounds in *D. peruviana* essential oil were α-phellandrene, limonene, and α-pinene, with the three compounds reaching approximately 83% of the total composition. Tolerance studies showed high biocompatibility of this essential oil with human keratinocytes. In vivo studies demonstrated a moisturizing effect and an alleviation of several events occurred during the inflammatory process after topical treatment with *D. peruviana* essential oil such as decline in skin edema; reduction in leukocytic infiltrate; and decrease in inflammatory cytokines TNFα, IL-8, IL-17A, and IL-23. Therefore, this essential oil could be an attractive treatment for skin inflammation.

## 1. Introduction

Acute inflammation occurs as a host immune response to external changes or cellular injury. Common signs of the inflammatory process are redness, heat, pain, and swelling [1]. The cutaneous immune system contains a diverse profile of inflammatory response mediators including antimicrobial peptides, complement proteins, and phagocytes such as neutrophils and macrophages [2]. This inflammatory process constitutes an essential defense mechanism to protect the integrity of the body against invaders including foreign substances, microorganisms, or cancer cells [3]. However, an inappropriate or excessive inflammatory response can mistakenly lead to damage of normal tissues, owing to a high production of reactive oxygen species, nitric oxide, and pro-inflammatory cytokines, which can trigger chronic inflammation implicated in the pathogenesis of skin disorders including psoriasis, dermatitis, and rosacea [3,4].

Volatile oils, essential oils (EOs) or simply essences, are the natural aromatic substances responsible for the fragrances of flowers, leaves, and other plant organs [5]. EOs are also known as volatile secondary metabolites, volatile compounds, or volatile fractions. EOs are especially abundant in the families Apiaceae, Asteraceae, Burseraceae, Lamiaceae, Myrtaceae, and Rutaceae [6]. Various functions are attributed to them in plants, such as protection against insects and herbivores and adaptation to water stress, and they are of great importance in pollination, as they constitute elements of chemical communication owing to their volatility and marked odor [5].

EOs can be extracted from flowers, fruits, leaves, roots, trunk, and other parts of the plant, and constitute values < 0.01% up to values > 3% of the dry weight of the plant [7]. In terms of their chemical composition, EOs are generally complex mixtures (from a few to more than 100 compounds) of highly variable constituents that belong almost exclusively to the group of terpenes and, to a lesser extent, to the group of aromatic compounds derived from phenylpropane (cinnamic aldehyde, eugenol, anethole, anisic aldehyde, and safrole, among others) [8]. The heterogeneity of essential oil (EO) compounds provides them with different biological properties, such as analgesic, anti-inflammatory, antioxidant, bactericidal, fungicidal, insecticidal, larvicidal, and repellent action, among others [9].

*Dacryodes* Vahl is a genus of small or large trees, with indehiscent fruits, with an oily mesocarp and a cartilaginous and smooth pyrene. The species of this genus are distributed in the tropical forests of Africa, America, and Asia. The generic name is from the Greek dakruon meaning “tear, drop”, referring to how resin droplets form on the bark surface. *Dacryodes peruviana* (Loes.) H.J. Lam is a native species from Ecuador known as “copal”, “copal comestible”, and “anime” (Spanish language); “wigonkawe”, “wiñimonkawe”, “winkayamogeinka”, and “witakeño” (Wao tededo, dialect of the Amazon region); “kunchay” and “wichilla kupall” (Kichwa language); “ccovi shasha” and “shasha” (A’ingae language, spoken by the Cofán people); and “kunchai” and “shiríkip” (Shuar chicham language) [10,11]. This species is widely distributed in the Amazonian and Andean Ecuadorian regions between 0 and 2500 m a.s.l.; in this country, it can be found especially in the Amazonian provinces of Morona-Santiago, Napo, Pastaza, and Zamora-Chinchipe [12].

The *Dacryodes peruviana* plant is a 20–25 m tall tree that produces fruits annually. Copal fruits are smooth, ovoid-shaped, greenish-red capsules, averaging 3.3 cm long (2 to 4 cm) and 1.25 cm in diameter, with a thin pericarp about 0.4 cm thick. The fruits open in three or four valves and contain one to four seeds approximately 1.5 cm long. The fruit is used as food for birds and monkeys. In the Ecuadorian Amazon, the seeds and the mesocarp of the fruit are edible, raw, and passed in hot water or on the coals. The resin of this species is used as mosquito repellent and as incense, as well as to smoke around the houses to eliminate evil spirits, and is smoked to treat the “mal aire” (mythical disease) [11]. The EO of copal fruits collected in the Ecuadorian Amazon exerted a moderate activity against Staphylococcus aureus and a repellent activity class 4 against mosquitoes (Diptera: Culicidae) at concentrations of 3%, 2%, and 1%, and class 3 for a concentration of 0.5%, and the same EO showed a weak antioxidant activity through the DPPH and ABTS methods [7].

Currently, Ecuador occupies the sixth position worldwide in the number of plant species per unit surface area, in 0.17% of the planet’s land surface hosts’ some 20,000 species, which makes this country a biodiversity hotspot [13]. However, the fact that there are few studies of its aromatic plant species, especially of the aromatic species of the Burseraceae family, and that anti-inflammatory activity of the *Dacryodes peruviana* EO has not been previously reported in the literature, have stimulated our interest in investigating the EO extracted from this species. Considering these remarkable findings, the aim of this research was to evaluate the anti-inflammatory activity of the *D. peruviana* fruit EO (*D. peruviana* EO).

## 2. Results

### 2.1. Essential Oil Isolation

Through hydrodistillation in a Clevenger-type apparatus, 240 mL of EO was obtained from 5 kg of *D. peruviana*, which represents a yield of 4.5 ± 0.3% (*v/w*) or 45 mL/Kg, considered as high yields, which makes the *D. peruviana* EO suitable for industrial applications.

### 2.2. Physical Properties of Essential Oil

The fruits of *D. peruviana* provided an EO with a density of ρ^20^ = 0.8456 ± 0.0023 g/cm^3^, refractive index of n^20^ = 1.4751 ± 0.0002, and specific rotation of [α]^20^ = +12.2 ± 0.7, as previously published [7].

### 2.3. Essential Oil Compounds’ Identification

The identification of volatile compounds present in *Dacryodes peruviana* EO was carried out by means of GC-FID and GC-MS using capillary nonpolar column DB-5MS. The results obtained are summarized in Table 1. In *D. peruviana* EO, twenty-four chemical constituents were identified, representing 99.78% of the total composition.

It was determined that the vast majority (97.48%) of *D. peruviana* EO compounds are monoterpene hydrocarbons (MHs) in nature; furthermore, a low number of oxygenated monoterpenes (OMs, 1.63%) and sesquiterpene hydrocarbons (SHs, 0.67%) were identified. The presence of oxygenated sesquiterpene (OS) and diterpenes was not determined. The principal constituents (>5%) are found to be MH α-phellandrene (CN: 7, CF: C10H16, MM: 136.13 Da) with 52.35 ± 3.14%, limonene (CN: 11, CF: C10H16, MM: 136.13 Da) with 22.51 ± 1.68%, α-pinene with 8.45 ± 0.63%, and ρ-cymene with 5.24 ± 0.56%.

### 2.4. Tolerance Studies: Cytotoxicity Assay

**Figure 1:**
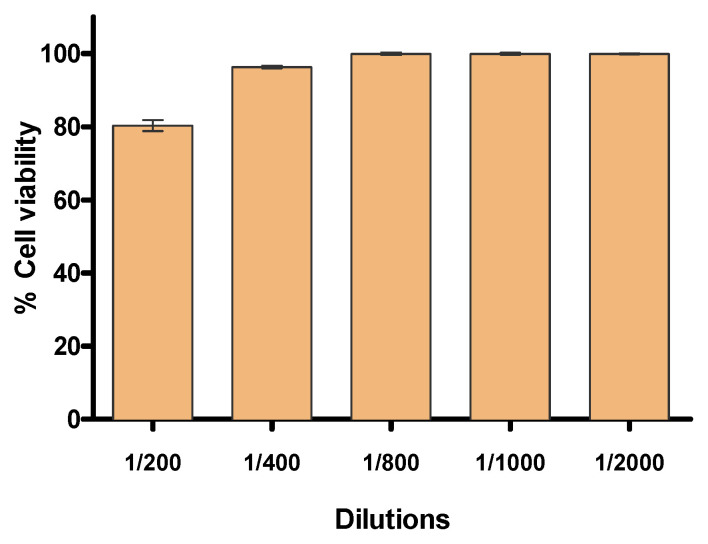
Percentage of cell viability of the HaCaT keratinocyte cell line exposed to different concentrations of *D. peruviana* EO.

Figure 1 shows the results of the MTT cytotoxicity assay using human keratinocytes HaCaT cell line. After 24 h of incubation, cell viability greater than 80% was observed in the assayed dilutions from 1/200 to 1/2000 (*v/v* in phosphate buffered solution—PBS). Dilutions from 1/400 showed cell viability close to 100% in relation to the control. Therefore, these results suggest that *D. peruviana* EO does not cause toxicity in human keratinocytes.

### 2.5. In Vivo Anti-Inflammatory Efficacy Studies: Arachidonic Acid (AA)-Induced Inflammation

#### 2.5.1. Stratum Corneum Hydration (SCH) and Thickness Evaluation

**Figure 2:**
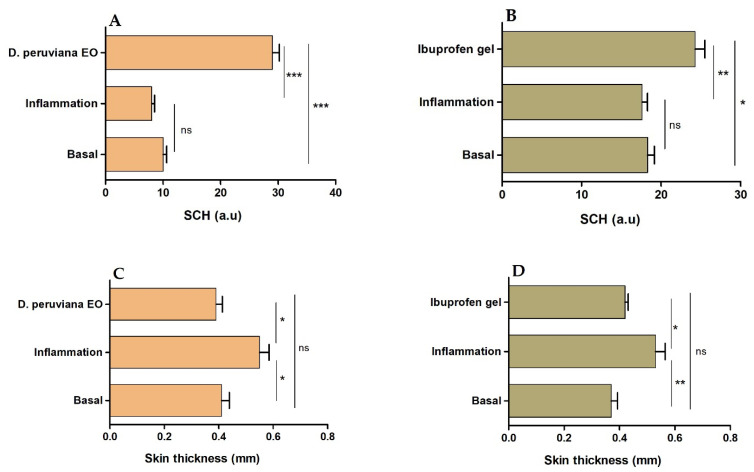
Stratum corneum hydration (SCH) and skin thickness of the mouse ears. (**A**) SCH of skin treated with *D. peruviana* EO, (**B**) SCH of skin treated with reference drug (Ibuprofen gel), (**C**) thickness of skin treated with *D. peruviana* EO, and (**D**) thickness of skin treated with reference drug (Ibuprofen gel). Mean ± SD of three replicates. Statistically significant difference: * = *p* < 0.05, ** = *p* < 0.01, *** = *p* < 0.001, ns: no significant.

Anti-inflammatory activity of *D. peruviana* EO was evaluated using a mouse model of inflammation induced by topical application of araquidonic acid (AA). After inducing inflammation, a slight decrease, but without significant statistical differences with respect to the basal state, was observed in the SCH levels. However, topical treatment with *D. peruviana* EO and ibuprofen gel on mice ears induced a significant increase in the skin hydration, ever higher than the basal state. The thickness of mice ears was markedly greater after inducing inflammation, whereas the treatment with *D. peruviana* EO and ibuprofen gel significantly reduced this parameter to the basal state and consequently alleviated the skin edema. Figure 2 shows the results for the stratum corneum hydration (SCH) and the variations in the ear thickness, representing the inflammatory process and the inflammatory reduction.

#### 2.5.2. Histological Analysis

**Figure 3:**
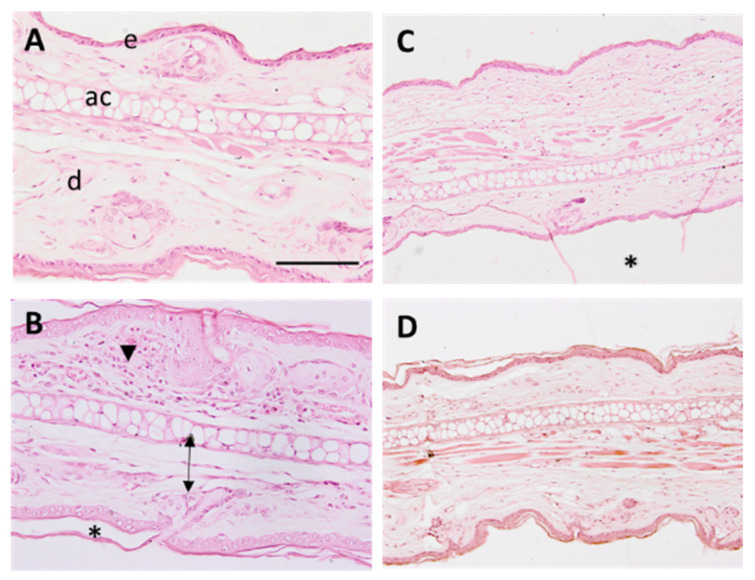
Micrographs of mouse ear samples (×100 magnification). (**A**) Negative control, (**B**) positive control, (**C**) reference drug (ibuprofen gel), and (**D**) treatment with *D. peruviana* EO. Scale bar = 200 µM. e: epidermis, d: dermis, ac: auricular cartilage. Arrowhead indicates neutrophilic infiltrates, arrows show edema, and asterisks indicate stratum corneum loss.

The histomorphological analysis of the mice ears was performed to assess the anti-inflammatory activity of the *D. peruviana* EO. Representative images of assayed treatments are shown in Figure 3.

A mild inflammation was observed in ears treated with AA (Figure 3B), characterized by a slight edema, increased leucocytic infiltrate, and loss of stratum corneum. The treatment with *D. peruviana* EO (Figure 3D) prevented the appearance of those inflammatory indicators and showed similar morphology as in negative control (Figure 3A) and treatment with ibuprofen gel (Figure 3C).

#### 2.5.3. Pro-Inflammatory Cytokines’ Determination

**Figure 4:**
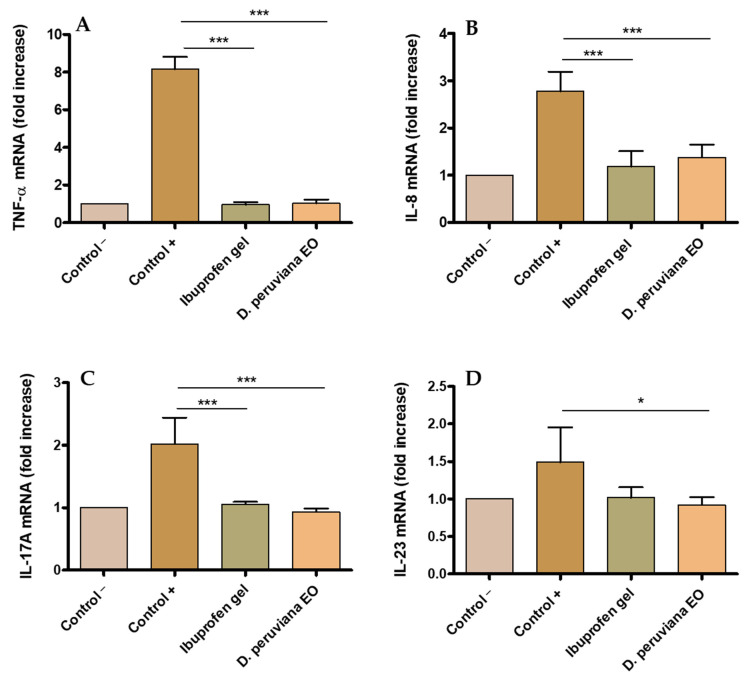
Expression of pro-inflammatory interleukins obtained by quantitative reverse transcription polymerase chain reaction (RT-qPCR): (**A**) tumor necrosis factor-alpha (TNF-α); (**B**) interleukin-8 (IL-8); (**C**) interleukin-17A (IL-17A); and (**D**) interleukin-23 (IL-23). Negative control (control −), positive (control +), treatment with reference drug (Ibuprofen gel), treatment with *D. peruviana* EO. Results are shown as mean ± SD (*n* = 3). Statistically significant difference: * = *p* < 0.05, *** = *p* < 0.001.

A statistically significant increase in the expression of cytokines TNF-α, IL-8, IL-17A, and IL-23 was observed in the positive control as result of the inflammation produced by AA when compared with the negative group. Topical treatment with *D. peruviana* EO on the inflamed area significantly reduced the expression of these pro-inflammatory cytokines to comparable levels to the negative control and with the ibuprofen gel treatment. These results suggest that *D. peruviana* EO could be acting in the regulation of inflammatory processes (Figure 4).

## 3. Discussion

The EO yield of *D. peruviana* fruit, 45 mL/Kg, is considered as high yield (>10 mL/Kg), according to the categorization proposed by the CYTED (Science and Technology for Development) [14]. The excellent yield of this species, added to the fact that the fruits are used instead of the wood, make the *D. peruviana* fruit a suitable option for use at industrial level. The predominant compounds in the chemical composition of *D. peruviana* EO were α-phellandrene, limonene (mixture of D- and L-), and α-pinene, with the three compounds reaching approximately 83% of the total composition. More than 50% (~52%) of the EO of *D. peruviana* fruits is constituted by α-phellandrene; this compound with CAS 99-83-2 is a cyclic monoterpene, which has been found as the main, major, or minor compound in various EOs [15,16,17]. α-phellandrene has low toxicity in rats and minimal risk of irritation sensitization [18]. Some studies have shown that α-phellandrene, limonene (CAS 138-86-3), and α-pinene (CAS 80-56-8) have some biological properties and activities such as anticancer, antimicrobial, anti-inflammatory, antidepressive, anti-leishmania, antioxidant, antihyperalgesic, antinociceptive, insecticidal, gastroprotective, and neuroprotective [18,19,20]. EOs containing α-phellandrene as one of the main compounds present antiacetylcholinesterase, antidepressive, antioxidant, antimicrobial, antihyperalgesic, and repellent activity [15,17,21,22].

The tolerance of *D. peruviana* EO was analyzed by in vitro studies, which are useful to screen the tolerability of pharmaceutical products prior to the pre-clinical and clinical assays. Cell lines are widely used for this type of study owing to their easiness to cultivate and sensitivity to toxic irritation [23]. The results obtained in this study showed that the analyzed dilutions of *D. peruviana* EO did not provoke cytotoxic effects on human keratinocytes after 24 h of incubation, suggesting high biocompatibility of this EO for skin application.

The anti-inflammatory potential of *D. peruviana* was studied using an AA-induced edema model in mouse ear. Inflammatory processes are a complex and biologically natural pathophysiological response initiated by vascular tissues; it helps the body to defend itself against pathogens, possible cell damage, and irritant damage. Cutaneous inflammation increases skin thickness, causes its dryness, promotes infiltration of inflammatory cells, and stimulates the release of various inflammatory mediators [24]. Increased skin thickness is one of the first signs manifested during inflammation, which is indicative of several events including edema, increased vascular permeability, and proliferation of keratinocytes [25]. In this study, topical application of AA caused an increase of 34.15% in the skin thickness as a result of the inflammatory process, whereas topical treatment with *D. peruviana* EO reduced this parameter to the basal state, showing a similar result to the reference anti-inflammatory drug—ibuprofen gel (Figure 2C,D). Additionally, SCH was determined in order to analyze the skin barrier function. The results of this evaluation showed only a slight decrease in SCH after inducing inflammation; however, a moisturizing action was noticeable in mice treated with *D. peruviana* EO (Figure 2A). These results were corroborated by histological analysis that showed the presence of a slight edema, leukocytic infiltrate, and loss of stratum corneum in the positive control. Conversely, topical treatment with *D. peruviana* EO aided in the improvement of these signs (Figure 3).

Inflammation begins with the activation of Phospholipase A2, which degrades lipids in cell membranes, resulting in the release of arachidonic acid and other inflammatory mediators such as eicosanoids, serotonin, histamine, and interleukins [26]. Interleukins consist of a large group of proteins that can cause many reactions in cells and tissues by binding to high-affinity receptors on cell surfaces. The main function of interleukins is thus to modulate growth, differentiation, and activation during inflammatory and immune responses [27]. In the present study, TNF-α, IL-8, IL-17A, and IL-23 were evaluated. TNF-α is a primary inflammatory factor released in the skin after trauma, injury, or infection and triggers the expression of other pro-inflammatory cytokines including IL-6, IL-8, and IL-1β [28]. IL-8 is produced by several types of cells, including monocytes, fibroblasts, endothelial cells, keratinocytes, and chondrocytes. Previous studies have shown that IL-8 levels increased with stimulus such as irradiation in keratinocyte cultures [29]. IL-17A acts on epithelial and endothelial cells. The main effects of IL-17A are the release of proinflammatory cytokines specifically in allergic processes [30]. Macrophages and dendritic cells mainly synthesize IL-23 acting on T cells, causing the maintenance of IL-17-producing T cells, and it increases in the induction of chronic intestinal inflammation, in autoimmune diseases, cancer, and psoriasis [31]. In this research, the increase in the expression of cytokines TNF-α, IL-8, IL-17A, and IL-23 in mice ears inflamed by topical application was counteracted by *D. peruviana* EO treatment, which restored the mRNA values and, consequently, this EO reduced the production of these pro-inflammatory cytokines to values similar to those of the negative control and ibuprofen gel treatment (Figure 4 A–D). The restorative benefits of *D. peruviana* in the clinical, histological, and immunological levels of skin inflammation may be due to the mixture of substances present in this EO, specifically by α-Phellandrene (52.35%) and D-limonene (22.51%). Specifically, α-Phellandrene exerts an anti-inflammatory action through different mechanisms, such as the inhibition of neutrophil migration towards the site of inflammation and the decrease in the production of proinflammatory cytokines induced by TNF-α, preventing the release of inflammatory mediators in the area. It is reported that α-Phellandrene has the potential to be used for the treatment of inflammatory diseases, such as rheumatoid arthritis, osteoarthritis, and allergic diseases [32]. Our results are in line with other investigations carried out in EOs, in which a reduction in the pro-inflammatory cytokins was observed when they tested EOs from Pinus spp in murine macrophages. α-pinene and α-phellandrene were the major compounds found in Pinus EOs among 45 compounds identified [33]. On the other hand, previous studies have reported the role of D-limonene administered subcutaneously in the reduction in proinflammatory cytokines that produce dermatitis as well as inhaled D-limonene in the decrease in lung inflammation caused by allergies [34,35]. D-limonene reduces the expression of TNF-α, IL-1β, and IL-6, and has been suggested as a stimulator of the production of IL-10, which is a powerful anti-inflammatory cytokine [36,37]. D-limonene demonstrated anti-inflammatory effects in colitis by decreasing cytokines (NF-κB, TNF-α, IL-1β, and IL-6) when administered orally in rats [38]. Therefore, these results suggest that *D. peruviana* EO acts in different events occurring during inflammatory processes and support its possible use in the treatment of inflammatory skin diseases.

## 4. Materials and Methods

### 4.1. Materials

Alliphatic hydrocarbons standard (code M-TPH6 × 4-1ML and name Diesel Range Organics Mixture #2-GRO/DR) was provided from CHEM SERVICE (West Chester, PA, USA). Arachidonic acid sodium salt, dichloromethane (DCM), and sodium sulfate anhydrous were purchased from Sigma-Aldrich (San Luis, MO, USA). Components for histological analysis were purchased from Thermo Fisher Scientific (Waltham, MA, USA). HaCaT cell line was purchased from Cell Line Services (Eppelheim, Germany). Helium (gas carrier) was obtained from INDURA (Quito, Ecuador). Reagents for the MTT ((3-[4,5-dimethylthiazol-2-yl]-2,5 diphenyl tetrazolium bromide) assay were obtained from Invitrogen Alfagene (Carcavelos, Lisbon, Portugal). Reagents used for cell cultures were purchased from Gibco (Carcavelos, Lisbon, Portugal). Transcutol-P (diethylene glycol monoethyl ether) was purchased by Gattefossé (Saint-Priest, France). All chemicals were of analytical grade.

### 4.2. Plant Material

The *Dacryodes peruviana* fruits in state of maturation were collected in La Paz parish. This parish, belonging to the Yacuambi canton, is located north of the Zamora Chinchipe province, in the Ecuadorian Amazon. The collection coordinates were a latitude of 3°36′40″ S and longitude of 78°39′38″ W. Copal fruits were harvested between the months of February and April. The collection conditions were a temperature of 25 °C and a pressure of 0.87 atm and the fruits were transported in closed plastic containers at an average temperature of 20 °C. The botanical specimens were identified by Nixon Cumbicus at the herbarium of the Universidad Técnica Particular de Loja (HUTPL). A voucher specimen is preserved in the HUTPL.

### 4.3. Essential Oil Isolation

The fruits were processed fresh, immediately after arriving at the laboratory, between 3 and 4 h after being collected. The postharvest treatment consists of the separation of foreign material and degraded fruits. The EO of copal fruits was extracted according to the procedures described by Valarezo, et al. (2020) [7] with some modifications, for which two consecutive procedures are used, the release and the isolation. The release was carried out in a patented device called “Device for the release of EO from a plant matrix by crushing by immersion centrifugal force” Title No. PI-2022-012, for which whole fruits were placed in the device, with a 1:1 ratio (fruit: water), and crushed for 45 s, until EO was released. This first step was carried out because the epicarp does not allow the exit of the EO found in the mesocarp, which is why the epicarp must be broken in adequate conditions to avoid the loss or degradation of the EO. In addition, this procedure called release allows to increase the contact surface between the raw material and the steam (in the distillation phase), which decreases the extraction times. After release isolation was performed, the isolation of EO was carried out by hydrodistillation, for which a semi-pilot distiller Clevenger type of 80 L of capacity was used. The distillation was carried out for 2 h and the EO collected was dried with anhydrous sodium sulphate and stored in amber sealed vials at 4 °C until being used in the subsequent analysis.

### 4.4. Determination of Physical Properties of Essential Oil

The physical properties of the EO determined were density, refraction index, and optical rotation, according to the standard method AFNOR NF T 75-111, AFNOR NF T 75-112, and ISO 592:1998, respectively. An analytical balance (Mettler AC 100, Mettler Toledo, Columbus, OH, USA) and a pycnometer were used to determine the density, a refractometer (ABBE, Hamburg, Germany) for the refraction index, and an automatic polarimeter (AP-81, MRC, Holon, Israel) for optical rotation. All measurements were performed at 20 °C.

### 4.5. Determination of the Chemical Composition of Essential Oil

The compounds present in the EO were determined qualitatively and quantitatively using a gas chromatograph (GC) (model 6890N series, Agilent Technologies, Santa Clara, CA, USA) according to the procedures described by Valarezo, et al. (2021) [39], with minimal modifications to some parameters. In the case of qualitative analyses, the GC was equipped with a mass spectrometer (type quadrupole) detector (MS) (model Agilent series 5973 inert, Agilent Technologies, Santa Clara, CA, USA) and, for quantitative analyses, GC was coupled to a flame ionization detector (FID). In both cases, an automatic injector (Agilent 7683, Agilent Technologies, Santa Clara, CA, USA) in split mode and a nonpolar column DB-5 ms were used. The samples are prepared with a ratio of 1/100 (*v/v*) of EO/DCM and then injected with a split ratio of 1:50. The temperature ramp was 50 °C for 3 min, then 2.5 °C/min until 210 °C, and 3 min at this temperature. The injector temperature was 210 °C and 250 °C for both detectors. The retention index (IR) was determined based on the comparison of retention times of the EO compounds and of the aliphatic hydrocarbons of standard injection under the same conditions. The compounds were identified based on a comparison of mass spectrum data and IRs with those published in the literature [40,41].

### 4.6. Tolerance Studies: Cytotoxicity Assay

Human immortalized keratinocytes (HaCaT) obtained from ATCC were used for in vitro experiments. They were maintained in Dubelco’s modified Eagle’s medium (DMEM) supplemented with 10% of fetal bovine serum (FBS), 1% non-essential amino acids, 100 U/mL of penicillin, and 100 g/mL of streptomycin. To perform the assays, cells were incubated at 37 °C and 5% of CO2 until 80–90% of growth. Methylthiazolydiphenyl-tetrazolium bromide (MTT) assay was used to evaluate the cytotoxicity effect of *D. peruviana* EO on HaCaT cells. For this purpose, 2 × 10^5^ cells/mL were cultured in 96-well plates (Corning) for 2 days. Then, different dilutions of *D. peruviana* EO (1/100, 1/400, 1/800, 1/1000 and 1/2000) in PBS (*v/v*) were incubated for 24 h. Afterwards, keratinocytes were washed with PBS 1× (Thermofisher) and incubated with MTT reagent at a concentration of 5 mg/mL (Sigma Aldrich) during 2 h maintaining 37 °C and 5% of CO_2_. The media was aspirated and MTT purple crystals were dissolved with Dimethyl sulfoxide (DMSO). Cytotoxicity levels were measured by absorbance at 570 nm using an Infinity Tecan 200 Pro Microplate Reader. As control, non-treated cells were used. The results were determined as the percentage of cell viability relative to the control (100% viability).

### 4.7. Anti-Inflammatory Efficacy Studies: Arachidonic Acid (AA)-Induced Edema Model in Mouse Ear

#### 4.7.1. Study Protocol

In vivo studies were carried out in accordance with the Bioethics Committee of the University of Barcelona (CEEA/UB ref. 4/16 and Generalitat ref. 8756. Date: 28 January 2016) using male BALB/c mice (*n* = 12, 4–5 months old). The inflammatory process was induced topically by applying 60 μL AA (at a concentration of 5 mg/mL diluted in PBS) on the right ears. A group of three inflamed animals were used as positive control. A second group (*n* = 3) was treated with 60 μL of *D. peruviana* EO 5% dissolved previously in Transcutol-P/water (1:1, *v/v*) after 20 min of AA application (*D. peruviana* EO group), whereas a third group (*n* = 3) was treated with a known anti-inflammatory drug (60 mg of ibuprofen gel 50 mg/g; reference: 886192.7). Transcutol-P was used to dissolve *D. peruviana* EO owing to its high skin biocompatibility. Finally, a group of untreated healthy mice (*n* = 3) was used to compare the results (negative group). The animals were sacrificed by cervical dislocation after 20 min and the right ears were cut in order to perform histological analysis and to evaluate the expression of pro-inflammatory cytokines.

#### 4.7.2. Stratum Corneum Hydration (SCH) and Thickness Evaluation

SCH and skin thickness of the mice ears were determined before the inflammation (basal state), after inflammation with AA, and after treatment with *D. peruviana* EO or ibuprofen gel. Corneometer CM-825 (Courage & Khazaka Electronics GmbH, Köln, Germany) and a digital thickness gauge of 0–10 mm (Mitutoyo Corp, Kawasaki, Japan) were used to measure these parameters, respectively.

#### 4.7.3. Histological Analysis

Histological evaluation was carried out by hematoxylin and eosin staining. To that end, the samples of mice ears were rinsed with PBS (pH 7.4) and stored overnight in 4% buffered formaldehyde. The tissues were dehydrated, embedded in paraffin wax, cut into transversal sections of 5 µm, and stained with hematoxylin and eosin. Finally, these samples were mounted on DPX (Sigma Aldrich, St. Louis, MO, USA) and observed under light microscope (BX41 microscope and XC50 camera, Olympus Hamburg, Hamburg, Germany).

#### 4.7.4. Pro-Inflammatory Cytokines Determination

TRI-Reagent^®^ (Sigma Aldrich) was used to extract total RNA from small fragments of mouse ear tissue. Purity and RNA concentration were measured on the NanoDrop One spectrophotometer (Thermo Scientific, Waltham, MA, USA) by the absorbance ratio at 260 and 280 nm. RNA was reverse-transcribed using the cDNA Reverse Transcription Kit (Thermo Fisher Scientific, Waltham, MA, USA) and the ProFlex PCR System (Applied Biosystems, Waltham, MA, USA). RT-qPCR reactions were carried out on the QuantStudio 7 Flex Real-Time PCR System (Applied Biosystems) using SYBR Green PCR Master Mix (Applied Biosystems) and cell-type specific primers for IL-17A, IL-8, IL-23, and TNFα (Table 2). The formula 2^−ΔΔCt^ was used to normalize the expression results. The values of the housekeeping GAPDH gene were used to standardize the values obtained for the studied genes.

#### 4.7.5. Statistical Analyses

These experiments were carried out in triplicate. The results are presented as the mean ± SD. Statistical analyses were performed by one-way ANOVA followed by Tukey’s test using the GraphPad Prism, v5.0 software (GraphPad Software Inc., San Diego, CA, USA). *p*-values less than 0.05 were considered statistically significant.

## 5. Conclusions

In conclusion, the present research provides evidence of the therapeutic benefits of that *D. peruviana* EO thanks to its content rich in terpenes, mainly α-phellandrene and limonene, in different aspects of skin inflammation including clinical, histological, and immunological. Its role in the inflammatory process was strongly demonstrated throughout the studies, showing a significant decline in edema, improvement in biophysical parameters (SCH), and reduction in leukocytic infiltrate as well as in the expression of proinflammatory cytokines including TNF-α, IL-8, IL-17A, and IL-23. Therefore, this EO could be used as a promising therapeutic treatment of local inflammation via topical application on affected area.

## Figures and Tables

**Table 1:** Chemical composition of *Dacryodes peruviana* essential oil.

CN	Rt	Compound	RI	RIf	%	SD	Type	CF	MM (Da)
1	5.83	α-Thujene	926	924	1.18	0.05	MH	C_10_H_16_	136.13
2	6.08	α-Pinene	932	932	8.45	0.63	MH	C_10_H_16_	136.13
3	6.7	Camphene	947	946	0.16	0.01	MH	C_10_H_16_	136.13
4	7.6	Sabinene	969	969	0.80	0.07	MH	C_10_H_16_	136.13
5	7.76	β-Pinene	973	974	3.39	0.05	MH	C_10_H_16_	136.13
6	8.29	Myrcene	986	988	0.82	0.02	MH	C_10_H_16_	136.13
7	9.07	α-Phellandrene	1005	1002	52.35	3.14	MH	C_10_H_16_	136.13
8	9.28	δ-3-Carene	1010	1008	0.08	0.01	MH	C_10_H_16_	136.13
9	9.4	α-Terpinene	1013	1014	0.31	0.03	MH	C_10_H_16_	136.13
10	9.73	p-Cymene	1021	1020	5.24	0.56	MH	C_10_H_14_	134.1
11	9.89	Limonene	1025	1024	22.51	1.68	MH	C_10_H_16_	136.13
12	11.12	γ-Terpinene	1055	1054	0.12	0.01	MH	C_10_H_16_	136.13
13	12.23	Terpinolene	1082	1086	2.08	0.17	MH	C_10_H_16_	136.13
14	14.61	Camphor	1140	1141	0.35	0.03	OM	C_10_H_16_O	152.12
15	15.84	Terpinen-4-ol	1170	1174	0.17	0.01	OM	C_10_H_18_O	154.14
16	17.15	γ-Terpineol	1202	1199	0.98	0.08	OM	C_10_H_18_O	154.14
17	18.54	Ascaridole	1236	1234	0.13	0.01	OM	C_10_H_16_O_2_	168.12
18	22.73	δ-Elemene	1338	1335	0.06	0.00	SH	C_15_H_24_	204.19
19	23.87	α-Copaene	1366	1374	0.08	0.01	SH	C_15_H_24_	204.19
20	25.88	trans-Caryophyllene	1415	1417	0.21	0.02	SH	C_15_H_24_	204.19
21	27.36	α-Humulene	1451	1452	tr		SH	C_15_H_24_	204.19
22	28.38	Germacrene D	1476	1480	0.13	0.01	SH	C_15_H_24_	204.19
23	29.74	δ-Amorphene	1509	1511	0.05	0.00	SH	C_15_H_24_	204.19
24	29.94	β-Curcumene	1514	1514	0.14	0.01	SH	C_15_H_24_	204.19
Monoterpene hydrocarbons (MH)					97.48				
Oxygenated monoterpenes (OM)					1.63				
Sesquiterpene hydrocarbons (SH)					0.67				
Total identified					99.78				

CN: compound number; Rt: retention time; RI: calculated retention indices; RIf: references retention indices; SD: standard deviation; CF: chemical formula; MM: monoisotopic mass; tr: traces (<0.5%); MH: monoterpene hydrocarbons; OM: oxygenated monoterpenes; SH: sesquiterpene hydrocarbons.

**Table 2:** Primer sequences used for the RT-qPCR assay.

Gene	Primer Sequence (5′ to 3′)	Gene Accession Number
GAPDH	FW: AGCTTGTCATCAACGGGAAG	BC023196.2
	RV: TTTGATGTTAGTGGGGTCTCG	
IL-8	FW: GCTGTGACCCTCTCTGTGAAG	X53798.1
	RV: CAAACTCCATCTTGTTGTGTC	
IL-17A	FW: TTTTCAGCAAGGAATGTGGA	NM_010552.3
	RV: TTCATTGTGGAGGGCAGAC	
TNFα	FW: AACTAGTGGTGCCAGCCGAT	NM_013693.3
	RV: CTTCACAGAGCAATGACTCC	

GAPDH = glyceraldehyde–3–phosphate dehydrogenase; IL–8 = interleukin-8; IL–17A = interleukin-17A; TNFα = tumor necrosis factor alpha; FW = forward primer; RV = reverse primer.

## Data Availability

Not applicable.
